# Cost effectiveness of using Faecal Immunochemical Testing (FIT) as an initial diagnostic investigation for patients with lower gastrointestinal symptoms suggestive of malignancy

**DOI:** 10.1186/s12875-021-01435-7

**Published:** 2021-05-12

**Authors:** CC Kearsey, C Graham, HS Lobb, J Chacko, R Weatherburn, PS Rooney

**Affiliations:** 1 University of Liverpool, Liverpool, UK; 2 Department of Coloproctology, Halton Teaching Hospitals NHS Foundation Trust, Warrington, UK; 3 Bristol Royal Infirmary, Bristol, UK; 4 Department of Colorectal Surgery, Royal Liverpool University Hospital, Liverpool, UK

**Keywords:** Colorectal Neoplasms, Models, Econometric, Referral and Consultation, Diagnostic Tests, Routine

## Abstract

**Background:**

There has been an increase in the numbers of patients presenting to primary care with suspected colorectal malignancy and subsequently an increase in demand for endoscopy. This study aims to forecast the cost of faecal immunochemical testing (FIT) compared to conventional diagnostic tests as a primary investigation for patients with symptoms suggestive of colorectal malignancy.

**Methods:**

Retrospectively, 1950 patients with symptoms suggestive of colorectal malignancy who were referred through primary care and underwent investigations through standard endoscopic evaluation were included. These patients were used to forecast the cost of faecal immunochemical testing creating theoretical data for sensitivity and specificity. Outcome measures included: the number of investigations under current protocol; cost of current investigations; number of predicted false negatives and false positives and positive/negative predictive values using current sensitivity data for FIT; the cost forecast of using FIT as the primary investigation for colorectal malignancy.

**Results:**

Median age was 65 (IQR 47–82) with 43.7% male and 56.3% female. A total of 1950 investigations were carried out with a diagnostic yield of 26 cancers (18 colon, 8 rectal), 138 polyps and 29 high risk adenomas (HGD ±  > 10 mm). In total, £713,948 was spent on the investigations. The commonest investigation was colonoscopy totalling £533,169. The total cost per cancer diagnosis was £27,459. Sensitivity (92.1% CI 86.9–95.3) and specificity (85.8% CI 78.3–90.1) for FIT in colorectal cancer was taken from NICE and was costed via the manufacturer(s). The projected total cost of FIT for the same population using a ≥ 4 μg haemoglobin cut off was £415,680 (£15,554 per cancer). The total cost of high-risk polyps using ≥ 4 μg cut off was £404,427 (sensitivity 71.2% CI 60.5–87.2, specificity 79.8%CI 76.1–83.7) or £13,945 per polyp.

**Conclusions:**

FIT is a cheaper and effective alternative test with the potential to replace current expensive methods. The forecast is based on the limited data available for sensitivity/specificity in the current literature. FIT has now been commenced for symptomatic patients in the UK and therefore sensitivity may change in the future.

**Supplementary Information:**

The online version contains supplementary material available at 10.1186/s12875-021-01435-7.

## Introduction

Faecal Immunochemical Test (FIT) is a quick faecel immunochemical test for Hb, designed to identify serious colorectal pathology. The test identifies small quantities of blood in the patient’s faeces (faecal occult blood (FOB)) using dedicated polyclonal antibodies to haemoglobin (Hb). Most colorectal pathology with the potential to become malignant tends to bleed more than native tissue. Therefore, the presence of blood in the faeces potentially indicates the presence of disease in the colon and/or rectum. Those who receive a positive FIT result precede to endoscopic investigation [1].

Currently, the standard protocol for patients presenting with symptoms associated with the lower gastrointestinal tract is investigation with endoscopy, thereby creating a large volume of requests for these services. A colonoscopy is an expensive, invasive procedure with significant risks and patient experience is often unfavorable [2].

Secondary to increase in demand, endoscopy departments are struggling to meet the needs of the current referral system and it has been found many colonoscopies are being performed on large quantities of patients who are cancer free, questioning the need for change [3].

NICE Guidance DG30 [4] now recommends FIT should be used as a “straight to test” diagnostic tool for patients whose symptoms are suggestive of colorectal cancer. This paper is a cost forecast model for the use of FIT as an initial diagnostic tool prior to endoscopy or CT in symptomatic, two-week-wait (2WW) lower gastrointestinal (GI) referral patients.

## Materials and Methods

Retrospective data was collected on all patients who presented to primary care with lower GI symptoms suggestive of colorectal cancer who were urgently referred according to standardised 2WW criteria created by the Department of Health, UK and the National Institute of Health and Care Excellence (NICE) to Royal Liverpool University Hospital between March 2015 and November 2017. Demographic details and specific referral benchmarks from primary care were also documented.

Specialist nurses at the Royal Liverpool University Hospital who had received detailed training on the process triaged the referrals and categorised patients into different groups for investigation. Referral criteria can be seen in Additional file 1. All patients could qualify for more than one group. All patients who underwent investigation were sent bowel preparation by post with information about the colonoscopy procedure. Individual suitability for the test was confirmed on the day by assessment by the endoscopist on a ‘case by case’ basis.

One HM-JACKarc analytical system (Kyowa Medex/Alpha Labs) was The FIT method used for all samples. The analytical working range was 2–8000 µg Hb/g faeces (µg/g). The limit of detection of the assay is 2 µg/g and the limit of quantification was 100 µg/g. Positivity was determined at 4 µg/g and classified into amber (4-10 µg/g) and red (> 10 µg/g). FITTER guidelines for reporting were used according to Fraser et al. [5].

Patients with normal tests were immediately discharged. Any positive findings were then reviewed by senior clinicians and it was decided at this point whether the patient a) was discharged, b) was sent for further investigations or treatment, or c) brought to clinic to discuss findings. Any patients who were deemed to have either an equivocal diagnosis or positive cancer diagnosis were referred to the local multi-disciplinary team (MDT) meeting for further management.

From collected data, a reverse cost forecast analysis was performed using sensitivity/specificity methods by Westwood et al. [6]. This data was then used to estimate false positives, false negatives, the positive predictive value (PPV) and the negative predictive value (NPV) that would have likely been in our population of patients if we had used FIT as the primary investigation. These values were then used to forecast the cost of FIT compared to the current protocol using the following calculation: true negatives x cost of one FIT + (true positives x cost of colonoscopy) + (false positives x cost of colonoscopy) + (false negatives x cost of colonoscopy). Data was analysed and results were derived using SPSS 24 (IBM, Armonk, New York, USA).

## Results

A total of 1950 patients were triaged through our service with a sex bias of, male (43.7%) and female (56.3%). Patient demographics included in the study are displayed in Table 1 below.

**Table 1:** Patient demographics

Demographics	
*Total Number of Patients*	*1950*
*Number of Males*	*852*
*Number of Females*	*1098*
*Median Age (interquartile range)*	*65.00 (47–82)*
*Median Age for Males (interquartile range)*	*65.00 (49–76)*
*Median Age for Females (interquartile range)*	*65.00 (47–82)*
*Average Pathway Duration*	*63.01*
*SD for Pathway Duration*	*45.59*

**Fig. 1:**
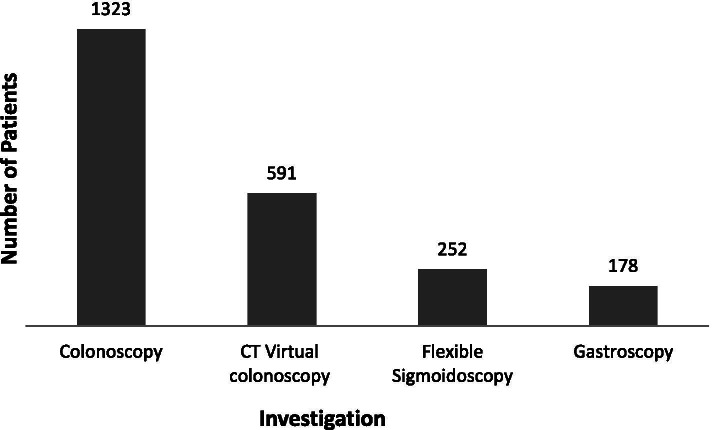
The distribution of investigations as per the current protocol

Median age of referral was 65 with a median local population age of 70. As expected, the main modalities of investigation were endoscopic (colonoscopy and flexible sigmoidoscopy) and radiological (CT colonography) with some patients undergoing aditional gastroscopy due to national guidelines for iron deficiency anaemia. The distribution of these investigations are shown in Fig. 1.

Patients were coded and categorised to one or more groups depending on their symptoms. The highest number of patients were found in the > 50 years group and with a change in bowel habit (looser faeces ± more frequent movements) (*n* = 754). There were no patients who coded into the < 50 years group with rectal bleeding and weight loss and no patients with a positive FOB screening referral. This was expected as they are investigated through a separate pathway.

**Fig. 2:**
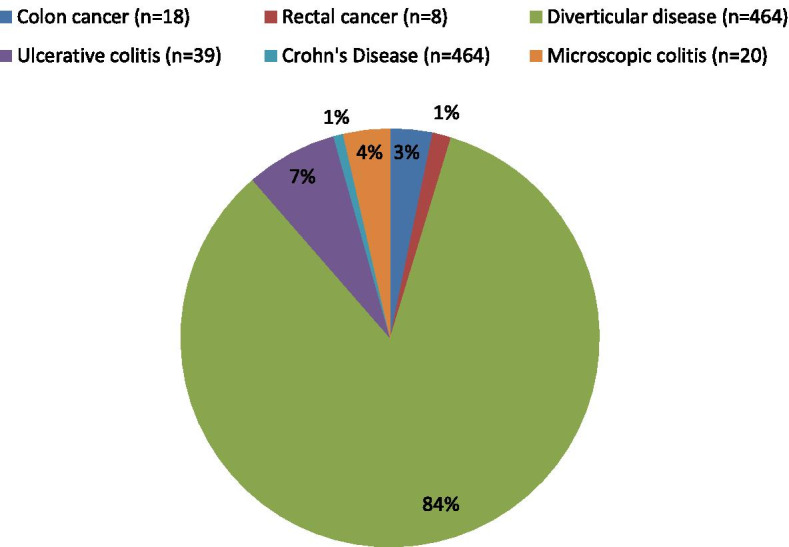
Distribution of diagnosis

The most common diagnosis was diverticular disease with a total of 474 patients. This was followed by ulcerative colitis (*n* = 39). A total of 26 cancer diagnoses were found, of which 18 (69%) were colon cancer and 8 (31%) were rectal cancer. There were 1323 patients with no diagnosis after investigation and were deemed normal/none-serious results and discharged. These figures are represented in Fig. 2.

**Fig. 3:**
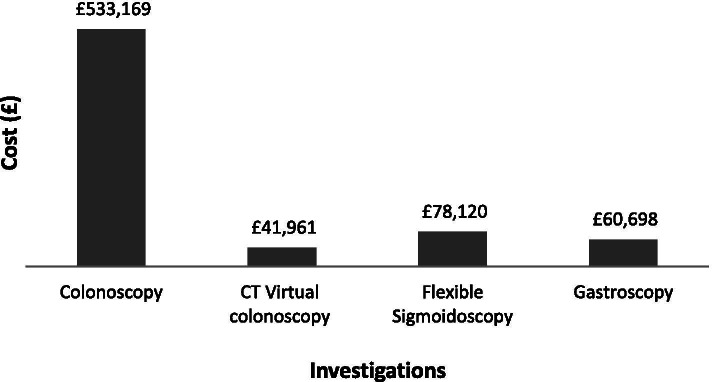
Cost breakdown of the investigations in the current protocol

A total of 1950 investigations were conducted which reported the finding of 26 cancers (approximately one cancer per 90 investigations) and 29 high-risk polyps (> 10 mm ± high grade dysplasia) resulting in a 2.7% yield of significant neoplasia. Figure 3 shows the total cost of investigations that took place. These were calculated using the tariffs released by the NHS for 2018/19. Naturally, the most significant cost is colonoscopy, which had the greatest number of patients (*n* = 1323) at a single test cost of £403 (£465 with biopsy). This was followed by CT colonography (*n* = 591) at a cost of £196 per scan. Flexible sigmoidoscopy (*n* = 252) cost £310 per test (£372 with biopsy) and finally gastroscopy (*n* = 178) at £341 per test (£388 with biopsy).

Tables 2 and 3 show the sensitivity and specificity of FIT at each of the Hb cut off values and the predicted false positives and false negatives modelling our population on FIT for CRC and high-risk polyps. The data was generated using the actual results obtained in the retrospective analysis and the predicted cost was calculated using the current sensitivity/specificity data used by NICE.

**Table 2:** FIT sensitivities/specificities at various Hb cut offs and modelling data for CRC

Hb Threshold	Sensitivity	95% CI	Specificity	95% CI	True +	True -	False +	False -	PPV (%)	NPV (%)
4 μg	*97.2*	*85.5–99.9*	*88.4*	*58.1–92.7*	*26*	*1895*	*258*	*0.8*	*2*	*99.9*
10 μg	*92.1*	*86.9–95.3*	*85.8*	*78.3–90.1*	*26*	*1895*	*314*	*2*	*7.5*	*99.8*
15 μg	*92.3*	*86.6–96.1*	*86.9*	*85.6–88.1*	*26*	*1895*	*286*	*2*	*8.2*	*99.8*
20 μg	*89.5*	*84.9–93.1*	*86.6*	*85.4–87.7*	*26*	*1895*	*293*	*3*	*8*	*99.8*

**Table 3:** FIT sensitivities/specificities at various Hb cut offs and modelling data for high-risk polyps

Hb Threshold	Sensitivity	95% CI	Specificity	95% CI	True +	True -	False +	False -	PPV (%)	NPV (%)
4 μg	*71.2*	*60.5–87.2*	*79.8*	*59.7–88.1*	*29*	*1895*	*503*	*11*	*6*	*99.2*
10 μg	*68.9*	*53.2–81.4*	*80.2*	*76.1–83.7*	*29*	*1895*	*468*	*13*	*7*	*99.2*
15 μg	*66.7*	*50.9–79.6*	*83.1*	*79.2–86.5*	*29*	*1895*	*385*	*14*	*7*	*99.3*
20 μg	*64.4*	*48.7–77.7*	*85.7*	*81.9–88.7*	*29*	*1895*	*316*	*16*	*8*	*99.1*

For the current protocol, £713,948 was spent in this study on investigations with colonoscopy costing £533,169 alone. The endoscopic/radiological evaluation for each cancer was £27,459 and £24,618 for high-risk polyps.

Depending upon manufacturer, FIT is significantly cheaper at £5 per test [7]. We forecasted the cost of using FIT as a primary investigation on our population using ≥ 4 μg Hb cut off with the following calculation (1895 x £5 + (true positives x £465) + (false positives x £465) + (false negatives x £465)). The total cost of FIT would have been £404,427, equating to £ 15,554 per cancer. The total cost of high-risk polyps using ≥ 4 μg cut off level was £13,945 per polyp.

## Discussion

Colorectal malignancy (CRC) is one of the top three most common cancers in the UK but is considered to be very treatable due to recent improvements in early detection [8]. FIT testing was originally developed as an advancement on guaiac Faecal Occult Blood testing in bowel cancer screening. It is designed to detect small amounts of blood in faeces using polyclonal antibodies to the Hb molecule [9]. Recent evidence has found with some certainty that the use of quantitative faecal immunochemical testing has substantial benefits over the FOB test in both accuracy and compliance [10]. This posed the question of whether FIT could be used as a primary diagnostic test rather than only for screening.

With rising awareness of CRC in the population, there has been an increase in the numbers of patients presenting to primary care with symptoms suggestive of CRC. This, coupled with a change in NICE guidance, resulted in more referrals to secondary care for 2WW cancer referrals and so in turn escalated the demand for colonoscopies [11].

If the current trend continues, Bowel Cancer UK estimates that there will be an increase in demand of 10–15% per year for diagnostic colonoscopies [8]. Endoscopy services are already stretched to capacity with the increasing demand and many patients breach the recommended 2WW for investigation. Secondly, endoscopic evaluation of the bowel is not without risk and carries a false negative rate of approximately 5% [12]. In addition, endoscopies are expensive and can be an unpleasant experience for patients.

In an attempt to streamline the referral system, initiatives have been created in some units such as nurse led colorectal telephone assessment pathways and the so called ‘straight to test’ pathway. These systems have been shown to reduce the time it takes for investigations to be carried out, but studies have revealed that patients may still be subject to inappropriate investigations for their symptoms, especially due to a lack of early assessment [13]. Thus, the identification of a cheaper, well tolerated investigation that has a robust sensitivity is of upmost importance. FIT has the potential to be used at the point of referral from primary care to guide the need for further investigations, reduce unnecessary colonoscopies and create a more cost-effective system. Despite this, it is important to consider the detection and removal of polyps during colonoscopy which reduces future CRC incidence and mortality.

When looking at Hb cut off values in faecal immunochemical testing, increasing the Hb cut-off decreases the sensitivity, decreases the specificity of the test and increases the number of false negatives. Using FIT with an Hb cut off of 4 μg Hb/g in our population, the number of false negatives would be 0.8. Although this is low, the acceptability of this rate on a larger scale must be considered. We forecast that any missed cancers would develop further symptoms and therefore represent at a later date. These theoretical patients with more advance disease have their own cost implications and more importantly the ethical implications associated with a missed cancer diagnosis. Robust safety netting advice for patients using FIT would be absolutely necessary.

Patients receiving negative FIT test results could be reassured they are unlikely to have cancer and discharged. Other possible outcomes of a negative FIT could be watch and wait, onward referral to colorectal outpatient clinic or repeat faecal immunochemical testing, particularly if the patient’s symptoms persist. It is vital that FIT results should not be viewed in isolation and clinical judgement remains of paramount importance. Results for high-risk polyps shows a reduced sensitivity/specificity and a higher false negative rate of 11 at the 4 μg Hb/g cut off and these would also have the possibility of missed malignancy.

Using a Hb cut-off of 4 μg Hb/g determined 258 false positives. This can be explained as FIT detects haemoglobin associated with a variety of other pathology such as inflammatory bowel disease [14]. Data from other studies suggested that up to 28.9% of patients with an initial false positive result from a FIT were eventually diagnosed with some form of serious bowel disease [15]. In our forecast, the patient who received a positive result would undergo colonoscopy as per the protocol and therefore patient disruption would be minimal.

Overall, the results of this study are positive and reveal a highly sensitive and specific test that could be used as a primary investigation for 2WW patients to facilitate the saving of colonoscopy resources. The cost of endoscopy to NHS England in 2014 was approximately £178.4 million and it was found that approximately 40% of tests were normal [16] which is comparable to our study. This study shows the cost of diagnosing colorectal cancer and high-risk polyps via endoscopy is significantly higher than the forecasted cost of FIT at £27,459 and £24,618 vs £15,554 and £13,945 respectively. This is a potential cost reduction of 43% over 2 years (£309,521).

## Conclusion

FIT is likely to be a cheaper alternative diagnostic than current methods with similar sensitivity and could potentially be used to replace the former, however, further studies pertaining to sensitivity and specificity are necessary.

## Supplementary Information

**Additional file 1.** Referral criteria by age and symptoms used by specialist nurses to determine appropriate investigation.

## Data Availability

The datasets used and/or analysed during the current study are available from the corresponding author on reasonable request.
