# Supplementary material for: Cost effectiveness of using Faecal Immunochemical Testing (FIT) as an initial diagnostic investigation for patients with lower gastrointestinal symptoms suggestive of malignancy
Source: BMC Fam Pract. 2021 May 12;22:90. doi: 10.1186/s12875-021-01435-7 (PMC8117514; doi:10.1186/s12875-021-01435-7)
Supplement: Supplementary file 1 — Additional file 1. Referral criteria by age and symptoms used by specialist nurses to determine appropriate investigation. [file 12875_2021_1435_MOESM1_ESM.docx]

| **Signs and Symptoms** | **Investigation** | **Other** |
| --- | --- | --- |
| **Age 40 and over** | | |
| Unexplained weight loss and abdominal pain | CT Colonography (CT ACE) |  |
| Change in bowel habit (depended on age and co-morbidity) | CT Colonography (CT ACE) |  |
| **Aged 50+** | | |
| Unexplained rectal bleeding (dark/changed blood; blood mixed with stool) | Colonoscopy |  |
| Explained minor rectal bleeding | Flexible Sigmoidoscopy |  |
| Change in bowel habit (looser stool +/- frequent stool) | Colonoscopy |  |
| **Any Age** | | |
| Rectal Mass | Flexible Sigmoidoscopy |  |
| Abdominal Mass | CT Colonography |  |
| FOB Positive | Colonoscopy |  |
| Anal Mass/Ulceration | Flexible Sigmoidoscopy +/- EUA |  |
| **Under 50 Years** | | |
| Rectal Bleeding and abdominal pain | Colonoscopy |  |
| Rectal Bleeding and change in bowel habit | Colonoscopy |  |
| Rectal Bleeding and weigh loss | Flexible Sigmoidoscopy +/- CT Scan |  |
| Rectal bleeding & IDA | Colonoscopy or CT Colonography +/- OGD (FBC, Ferritin, Iron Studies) |  |
| **Aged 60 and over** | | |
| Iron Deficiency Anaemia(FBC, Ferritin and Iron Studies) | Colonoscopy or CT Colonography +/- OGD |  |
| Constipation (Acute) | Flexible Sigmoidoscopy (TWR) | CT Colonography depended on age and co-morbidity |
| Constipation (Chronic) | Flexible Sigmoidoscopy (Routine) | CT Colonography depended on age and co-morbidity |
| Tenesmus | Flexible Sigmoidoscopy |  |
| Mucus | Flexible Sigmoidoscopy |  |
| Faecal incontinence | Flexible Sigmoidoscopy |  |

***Appendix 1: Referral criteria by age and symptoms used by specialist nurses to determine appropriate investigation***
